# Confinement, chaotic transport, and trapping of active swimmers in time-periodic flows

**DOI:** 10.1126/sciadv.add6196

**Published:** 2022-12-07

**Authors:** Boyang Qin, Paulo E. Arratia

**Affiliations:** ^1^ Department of Mechanical and Aerospace Engineering, Princeton University, Princeton, NJ 08544, USA.; ^2^ Department of Molecular Biology, Princeton University, Princeton, NJ 08544, USA.; ^3^ Department of Mechanical Engineering and Applied Mechanics, University of Pennsylvania, Philadelphia, PA 19104, USA.

## Abstract

Microorganisms encounter complex unsteady flows, including algal blooms in marine settings, microbial infections in airways, and bioreactors for vaccine and biofuel production. Here, we study the transport of active swimmers in two-dimensional time-periodic flows using Langevin simulations and experiments with swimming bacteria. We find that long-term swimmer transport is controlled by two parameters, the pathlength of the unsteady flow and the normalized swimmer speed. The pathlength nonmonotonically controls swimmer dispersion dynamics, giving rise to three distinct dispersion regimes. Weak flows hinder swimmer transport by confining cells toward flow manifolds. As pathlength increases, chaotic transport along flow manifolds initiates, maximizing the number of unique flow cells traveled. Last, strong flows trap swimmers at the vortex core, suppressing dispersal. Experiments with *Vibrio cholerae* showed qualitative agreement with model dispersion patterns. Our results reveal that nontrivial chaotic transport can arise in simple unsteady flows and suggest a potentially optimal dispersal strategy for microswimmers in nature.

## Teaser

Flow pathlengths non-monotonically control swimmer dispersion and provide optimal transport strategies in time-periodic flows.

## INTRODUCTION

From algae blooms in lakes and rivers to sperm cells in the reproductive tract, swimming microorganisms often encounter unsteady flows that span a wide range of length scales. Large oceanic flows such as temperature-driven meridional overturning circulations, commonly known as global conveyor belts, drive the global-scale transport of carbon-fixing marine microorganisms and nutrient flow (*1*–*3*). At smaller scales, local flow gradients exert forces and torques on microorganisms, which can alter swimmer motility and overall transport (*4*) and lead to intriguing physical phenomena not found for passive particles such as rheotaxis (*5*, *6*), gyrotaxis (*7*–*9*), and gradient-induced chemotaxis (*10*, *11*).

Microorganisms, in turn, may adapt their motility strategies, actively modify external flows, and take advantage of the coupling between motility and flow to forage and reproduce (*12*). Under quiescent flow conditions, for example, microswimmers can initiate collective fluctuating motions in the fluid medium via hydrodynamic interactions (*13*, *14*). In unsteady (time-periodic) flows, bacterial swimmers can attenuate large-scale scalar transport while enhancing mixing at small scales (*15*). These self-induced flows and flow modifications can lead to intriguing properties in fluids containing active swimmers, including enhanced Brownian diffusivity (*16*–*18*), active fluid transport and mixing (*19*–*21*), and, possibly, work extraction (*22*, *23*).

Even simple shear flows can profoundly alter the movement of microswimmers (*24*–*26*). Experiments show that motile bacteria can drift across streamlines out of the plane of shear (*6*), escape low-shear regions, and become trapped in high-shear regions (*24*). Motile phytoplankton are found to accumulate in or be depleted from regions of different shear rates (*25*), and they form cell assemblages called “thin layers” (*26*). The behavior of microswimmers in complex flows shows equally rich dynamics but is much less understood. In turbulent flows, for example, gyrotactic swimmers can cluster in small-scale patches (*8*, *27*) and gather in regions of positive velocity gradients (*28*). Numerical simulations in isotropic turbulence show that elongated swimmers preferentially align with flow velocity (*29*), while clustering and patchiness are greatly reduced (*30*). In chaotic flows, simulations show that rod-like swimmers can be trapped or expelled by elliptic islands (*31*), i.e., Kolmogorov-Arnold-Moser tori (*32*), depending on their shapes and swimming speeds. The trapping of particles in elliptic islands can lead to a reduction in swimmer transport (*33*, *34*). In steady convection rolls, steric interaction may lead to complex clustering patterns of active particles (*35*), while transient non-Gaussian diffusion is found at short observation times (*36*). Recently, bacterial swimmers are found to exhibit trapping and hopping dynamics in porous media where the optimal spreading is controlled by run length (*37*, *38*). Study of steady flows in porous media showed that bacteria align and accumulate along flow regions that experience large stretching (*39*). Despite recent progress, however, the dispersion dynamics and transport of active swimmers in unsteady complex flows remain poorly understood.

In this contribution, we conduct both Langevin simulations and bacterial dispersion experiments to investigate the long-term transport and aggregation dynamics of smooth active swimmers in time-periodic flows composed of an array of vortices. Although the displacement due to flow completely reverses itself in any given cycle, we find that the transport of microswimmers is substantially altered by the time-periodic flow. The flow time periodicity, when coupled to the flow topology and swimmer motility, gives rise to a series of swimmer dispersal regimes with highly nonmonotonic transport efficiency, not found in steady flow counterparts. A single flow control parameter, which we term the pathlength, dictates the nonmonotonic dispersion and gives rise to chaotic swimmer dynamics for swimmers with fixed speed. At moderate flow strengths, swimmer transport is hindered by as much as 10-fold compared to free swimming as swimmers are drawn toward the flow’s invariant structures (or manifolds) that form the boundaries of the vortices. As pathlength increases, we observe an enhancement in flow-induced swimmer transport along flow manifolds that is purely diffusive in nature. The number of unique flow cells traveled by the swimmer during dispersion is maximized in this regime, where swimmers gain access to the largest number of potential foraging grounds in the flow domain. Last, for very strong flows, limit cycles emerge within the vortex that trap a subset of swimmers and suppress dispersal; vortex trapping is stronger for slower swimmers. Our results reveal that the flow-induced dispersion is highly nonmonotonic with flow strength and shows distinct aggregation and dispersion mechanisms. Aggregation along flow manifolds and trapping in vortices observed here may alter swimmer encounter rates, nutrient gradients, and accumulation of signaling molecules. This complex coupling between swimmer behavior and flow structures and dynamics could lead to optimal dispersal strategies and distinct bacterial lifestyle transition patterns in natural and industrial mixing environments.

**Fig. 1.**
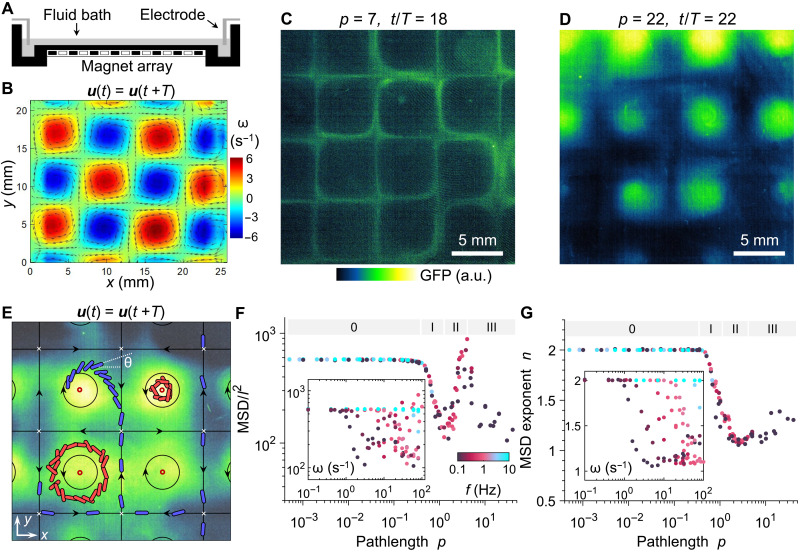
Dispersion patterns of active bacteria and long-term swimmer transport from the Langevin model reveal distinct dispersion regimes mediated by the time-periodic flow. (**A**) Schematic of the periodic flow cell apparatus used in the experiments. (**B**) Flow vorticity and velocity vectors in the experimental flow cell, obtained by particle tracking velocimetry. (**C** and **D**) Cell distribution patterns in bacteria dispersion experiments using *V. cholerae* labeled with green fluorescent protein (GFP) at (C) pathlength *p* = 7 and time *t*/*T* = 18 in the manifold trafficking regime and (D) pathlength *p* = 20 and time *t*/*T* = 2 in the vortex trapping regime. Colors indicate GFP intensity. a.u., arbitrary units. (**E**) Schematic of active swimmer transport in the time-periodic flow. Black arrows indicate directions of vortices and flow manifolds, which connect hyperbolic fixed points (white crosses). Fluorescent bacterial swimmers (green) are allowed to disperse under time-periodic flow. As flow pathlength increases, a subset of swimmers spiral outward the initial vortex and participate in chaotic transport along flow manifolds (blue, regime II). At high pathlength, increasing numbers of swimmers are trapped (red, regime III) near elliptic points (red circles). Swimmer orientation is indicated by the angle θ. (**F**) Ensemble-averaged stroboscopic MSDs of active swimmers as a function of flow pathlength *p* for various flow frequencies *f* and flow peak vorticities ω at time *t* = 2000 s, normalized by the flow quarter-cell length *l*^2^ for *u*_s_ = 80 μm/s. Inset: Identical MSD plotted against ω. (**G**) The corresponding MSD scaling exponent *n*. Inset: Identical exponent *n* plotted against ω. The designations 0, I, II, and III in (F) and (G) indicate transport regimes.

Experiments are performed in an electromagnetically driven fluid layer (*15*, *40*, *41*). A time-periodic current travels horizontally through a conducting fluid layer that is placed above an array of permanent magnets with alternating polarity (Fig. 1A). The resulting Lorentz forces drive a time-periodic cellular flow, namely, **u**(*t*) = **u**(*t* + *T*), that is spatially ordered due to the arrangement of the forcing magnets (Fig. 1B and fig. S1); the size of each vortex (or cell) corresponds to the spacing between magnets (~6.5 mm). We quantify the experimental flow field using particle tracking velocimetry (see Fig. 1B and Materials and Methods). The flow vorticity peaks at the elliptic fix points (or vortex center) of the flow; fluid stretching is maximized along the (stable and unstable) manifolds that separate neighboring vortices and connects the hyperbolic fixed points (*40*, *41*); to a first approximation, these manifolds are invariant flow structures that experience high levels of deformation (or strain). Bacterial dispersion experiments are conducted by adding a small amount of fluorescently labeled bacteria suspension at the center of a vortex cell. The suspension is then allowed to disperse in the flow and characterized using image analysis (see Materials and Methods). All experiments are performed at Reynolds numbers, *Re* = *Ul*/ν, ranging from 0.1 to 10, where ρ is fluid density, *U* is peak flow velocity, *l* = *L*/2 is the quarter-cell length of the vortex array, and ν is kinematic viscosity. Time-periodic flow with irregularly spaced forcing is known to produce chaotic mixing of passive scalars even at a low Reynolds number where inertial flow instabilities are absent (*40*, *41*). Flow characteristics can be adjusted by independently varying the imposed current and frequency. Last, we define *p =* ω/4π^2^*f*, which we term the flow pathlength. The pathlength *p* is a nondimensional parameter that characterizes the time-periodic flow and has been shown to modulate mixing in passive systems (*40*).

The model flow system in the simulation consists of a two-dimensional (2D) Taylor-Green vortex array that is periodic in space and time (fig. S1). Similar to the experimental flow, each vortex occupies a quarter-cell with a length of *l* = 6.5 mm and rotates in opposite direction to its immediate neighbors in a checkerboard pattern. The stream function of the flow oscillates sinusoidally in time with frequency *f*, period *T*, and amplitude *Ul*/π. Stochastic simulations of 10^4^ active swimmers are conducted for each flow condition where the translational diffusion is modeled as random Wiener processes. The active swimmers are modeled as noninteracting, smooth-swimming axisymmetric ellipsoids that move with velocity *u*_s_ and reorient in the flow gradients according to Jeffery’s dynamics (*42*) without rotational diffusivity unless stated otherwise. We note that rotational reorientation is often weak for fast-swimming bacteria (at short times), swimmers that lack run-and-tumble behavior or have impaired chemotaxis signaling pathways (*43*), and synthetic Janus-particle swimmers (*44*). Variations in swimming speed are nondimensionalized as *u*_s_*T*/*l* to reflect the swimmer motility in a flow period. The net swimmer velocity is the superposition of the underlying flow and the intrinsic swimming velocity (see Materials and Methods).

## RESULTS

### Emergence of distinct swimmer transport patterns is universally controlled by the flow pathlength

We begin with dispersion patterns of the Gram-negative bacterium *Vibrio cholerae* in the flow cell apparatus (see Fig. 1C and Materials and Methods) at different flow frequency and peak vorticity. *V. cholerae*, the causative agent for the highly contagious disease cholera, is an aquatic-dwelling pathogen that encounters unsteady flows in nature. The transport and dispersion of *V. cholerae* in unsteady flows may affect disease transmission in natural environments. The swimming speed of this uniflagellate swimmer ranges from 40 to 80 μm/s (*45*, *46*). Unexpectedly, while the flow displacement always reverses itself in a single period for all flow conditions, the overall transport patterns of active swimmers are markedly different. We use the flow pathlength *p* to describe the flow conditions for reasons that will be clear in later sections. At *p* = 7, the dispersion patterns of the fluorescently labeled bacteria (Fig. 1C) show strong aggregation along flow manifolds. At very high pathlengths, however, strong trapping of the bacteria in the vortex cores occurs (Fig. 1D).

To reveal the mechanism underlying the dispersion patterns observed in *V. cholerae*, we conduct Langevin simulations in vortex array similar to that used in the experiments (fig. S1). In the model time-periodic flow, vorticity is maximal at elliptic fixed points, whereas flow manifolds (vortex boundaries) connect hyperbolic fixed points (Fig. 1E). We begin by investigating the overall transport efficiency of active swimmers. The goal is to uncover the key parameters controlling long-term swimmer dispersion. Swimmer transport is characterized by the ensemble-averaged long-term mean squared displacements (MSDs) normalized by the size of the vortex *l*. The quantity MSD/*l*^2^ is computed for *t* = 2000 s, a sufficiently long time for the MSD to reach a statistically stationary slope (fig. S2). We first show MSD/*l*^2^ as a function of flow vorticity ω (0.1 to 100 s^−1^) and oscillation frequency *f* (0.1 to 10 Hz), as in the inset of Fig. 1F. Results indicate that the variation in MSD with ω is strongly scattered for different flow frequencies *f* and that reductions in transport occur at different ω values. However, when plotted against the ratio of these two parameters, namely, the pathlength *p* = ω/4π^2^*f*, the data collapse into a single master curve (Fig. 1F). The pathlength measures the maximum angular displacement experienced by a passive fluid particle in a flow cycle and arises from the nondimensional governing equation (Eqs. 4a to 4d). Similarly, the scattered variations in the MSD scaling exponent *n* as a function of ω (Fig. 1G, inset) also collapse when plotted against pathlength *p* (Fig. 1G). Hence, the angular displacement per cycle controls the overall strength and the nature of swimmer transport.

### Flow pathlength nonmonotonically controls four swimmer dispersion regimes

As the control parameter *p* increases, we find distinct regimes of active swimmer transport with highly nonmonotonic changes in dispersion efficiency. For the lowest flow strengths (*p* < 0.5), the swimmers behave essentially as “free swimmers.” The stroboscopic MSD/*l*^2^ is independent of *p* (regime 0, Fig. 1F), and the MSD scaling exponent is constant at *n* = 2 (regime 0, Fig. 1G). Hence, for *p* < 0.5, the weak oscillatory flow is insufficient to alter the ballistic free-swimming nature of microorganisms. As *p* increases (0.5 ≲ *p* < 2), however, we observe a drastic reduction in swimmer transport MSD by approximately one order of magnitude (regime I, Fig. 1F). Concurrently, the stroboscopic MSD scaling exponent *n* decreases to approximately 1.2, indicating a transition from ballistic to nearly diffusive transport behavior (regime I, Fig. 1G). We characterize this transport regime as “manifold confinement” (regime I) for reasons described below. As *p* further increases (2 < *p* ≲ 5), the trend in MSD reverses and increases sharply by nearly an order of magnitude (regime II, Fig. 1F). Unexpectedly, the MSD scaling exponent *n* remains close to 1.0 (regime II, Fig. 1G), indicating a unique mechanism of flow-enabled transport that is purely diffusive in nature. We characterize this transport regime as “manifold trafficking” (regime II). Last, at very high *p* (*p* > 5) or very large angular displacement per cycle, the MSD decreases again, while swimmer transport remains diffusive (regime III, Fig. 1, F and G). We characterize this transport regime as “vortex trapping.” The emergence of these distinct transport regimes occurs for a range of swimmer aspect ratios (fig. S3) and rotational diffusivities (fig. S4). These results indicate that increasing the flow strength via pathlength *p* enables the sensitive control of flow-induced swimmer dispersion in a nonmonotonic fashion.

### Distinct dispersion patterns underlie different swimmer transport regimes

**Fig. 2.**
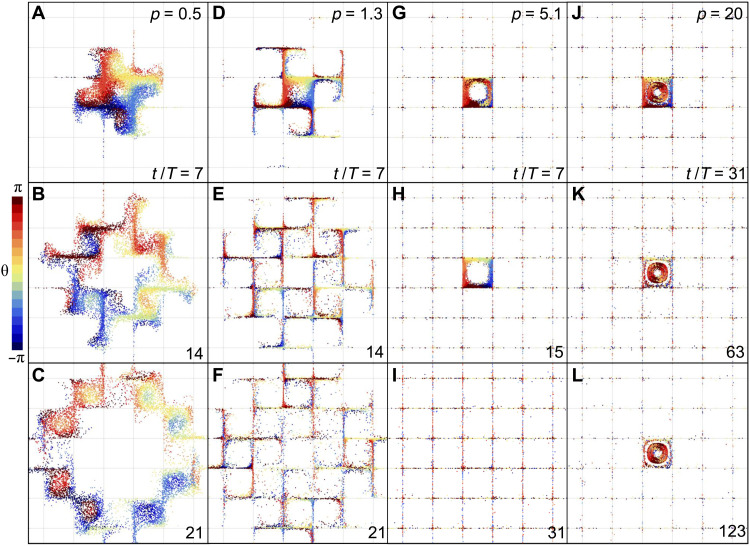
Dispersion pattern of active swimmers in time-periodic flows shows four distinct dispersion regimes controlled by a single flow parameter, the pathlength. (**A** to **L**) Swimmer spatial distribution from the Langevin model at the indicated time points normalized by the flow period *t*/*T*, colored by the corresponding swimmer orientation angles θ for (A to C) *p* = 0.5 at the start of the manifold confinement regime, (D to F) *p* = 1.3 at the minima of the normalized MSD in the manifold confinement regime, (G to I) *p* = 5.1 at the peak of the diffusive manifold trafficking regime, and (J to L) *p* = 20 in the vortex trapping regime. Swimmer speed is *u*_s_*T*/*l* = 0.12 for all cases.

Next, we explore swimmer dispersion dynamics and patterns underlying the nonmonotonic variations in long-term transport. The spatial distribution and corresponding swimmer orientations obtained from numerical simulations are shown in Fig. 2 (A to L) at various times during the dispersion process for each of the transport regimes described above. Before the onset of the manifold confinement regime (regime I, *p* = 0.5), where swimmer MSD begins to deviate from that of the free-swimming case, we find that swimmers propagate radially outward from the seeding quarter-cell (Fig. 2, A to C, and movie S1); swimmers show no diffusive random motion, leaving the initial quarter-cell devoid of swimmers after 21 flow cycles (Fig. 2C). Swimmer trajectories bend toward the vortices encountered in the swimming paths and penetrate the vortex cores. As *p* increases (regime I, *p* = 1.3), strong swimmer aggregation occurs toward flow manifolds or boundaries separating the flow quarter-cells (Fig. 2, D to F, and movie S2). Swimmers no longer travel unidirectionally outward, and they are unable to penetrate vortex cores. Rather, swimmer trajectories become highly random as they aggregate near flow manifolds, where many cells continue to occupy the seeding quarter-cell at *t*/*T* = 21. As *p* increases further (regime II, *p* = 5.1), swimmers are strongly aggregated and aligned with flow manifolds. Swimmer transport (MSD) is maximized, and the motion is dominated by the flow-induced transport along flow manifolds, allowing the swimmers to efficiently explore the flow domain in a diffusive fashion (Fig. 2, G to I, and movie S3); this is the manifold trafficking regime. Last, at *p* = 20 (regime III) and the highest flow pathlength, strong trapping of swimmers occurs at the initial vortex core where a subset of swimmers persist in the initial vortex (Fig. 2, J to L, and movie S4) despite hundreds of flow cycles. These dispersion patterns qualitatively agree with those found in experiments of bacterial swimmers (Fig. 1, C and D) in the manifold trafficking and vortex trapping regimes. Together, our results suggest that the transport of active swimmers in time-periodic flows is characterized by a series of flow-induced aggregation, dispersion, and trapping.

### Deterministic-to-chaotic transition in swimmer trajectories correlates with aggregation and alignment along flow manifolds

**Fig. 3.**
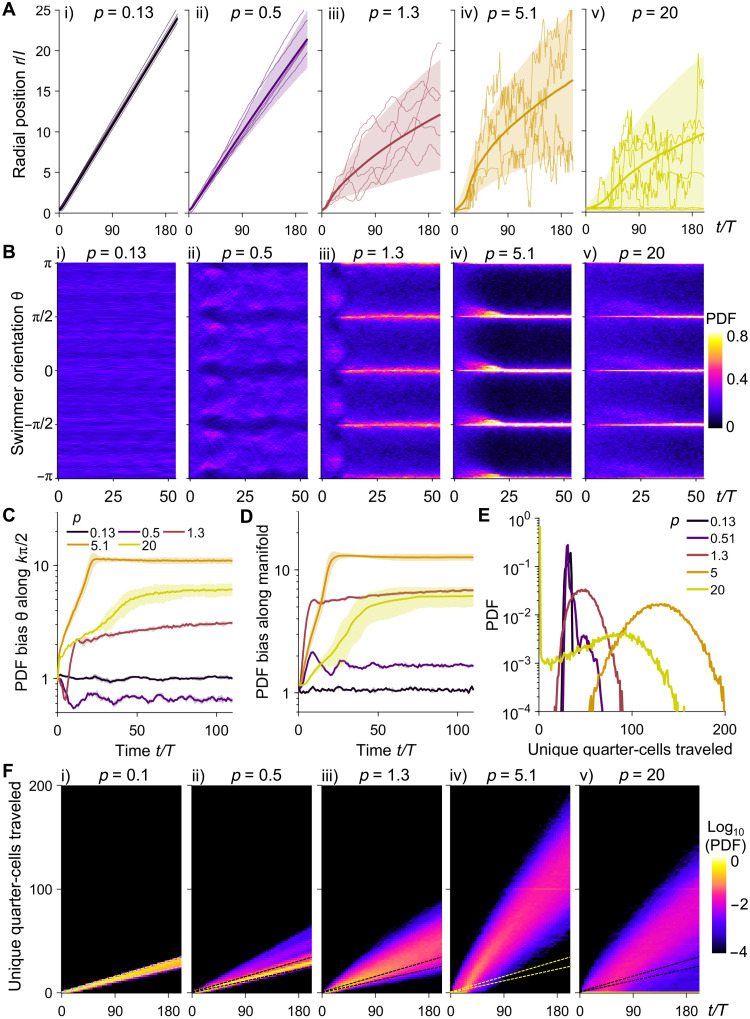
Swimmer radial spreading, swimming orientation bias, aggregation toward flow manifolds, and unique flow cells traveled are nonmonotonically controlled by the flow pathlength in the four dispersion regimes. (**A**) Time traces of radial positions *r* normalized by half flow cell period *l* for representative individual swimmers (thin line) and the population average (bold line) for (i) *p* = 0.13, (ii) *p* = 0.5, (iii) *p* = 1.3, (iv) *p* = 5.1, and (v) *p* = 20. Swimmer speed is *u*_s_*T*/*l* = 0.12 for all cases. Shaded regions indicate SDs from three replicates. (**B**) Time development of the PDF distribution of swimming orientation θ for (i) *p* = 0.13, (ii) *p* = 0.5, (iii) *p* = 1.3, (iv) *p* = 5.1, and (v) *p* = 20. Alignment with flow manifolds is indicated by θ = *k*π/2 where *k* = ±2, ±1, and 0. (**C**) Time development of the PDF bias of swimming orientation toward *k*π/2, *k* = ±2, ±1, and 0. The PDF bias is defined as the swimming orientation PDF along *k*π/2, normalized by that of a uniformly distributed swimming orientation. Shaded regions indicate SDs from three replicates. (**D**) PDF bias of swimmer aggregation toward flow manifolds, i.e., the boundaries between adjacent flow cells, defined as the PDF at flow manifolds, normalized by that of a uniformly distributed swimmer position. Shaded regions indicate SDs from three replicates. (**E**) PDF of unique flow quarter-cells traveled by the population of swimmers at *t*/*T* = 200 for various flow pathlengths. (**F**) Time development of the PDF of unique quarter-cells traveled for (i) *p* = 0.13, (ii) *p* = 0.5, (iii) *p* = 1.3, (iv) *p* = 5.1, and (v) *p* = 20. Dashed lines represent the boundaries of the PDF distribution for *p* = 0.1, i.e., for the free-swimming regime.

We now quantify swimmer aggregation, alignment, and dispersion statistics in each of the flow-induced transport regimes. For weak flows (*p* = 0.13, free-swimming regime), the ensemble-averaged radial position relative to the initial vortex center of the swimmers increases linearly in time (i, Fig. 3A) with minimal deviations of individual swimmers due to their smooth-swimming kinetics. The swimmer orientation angles are random and uniformly distributed in all directions during dispersion (i, Fig. 3B). At the onset of the manifold confinement regime (*p* = 0.5), the population radial displacement declines while the standard deviations (SDs) increase, showing larger variations between individual swimmers (ii, Fig. 3A). The swimmers avoid alignment with flow manifolds, as characterized by the lowered probability distribution at θ = *k*π/2, where *k* = ±2, ±1, and 0 (ii, Fig. 3B). As *p* further increases in the manifold confinement regime (*p* = 1.3), the population average radial displacements decrease sharply and transition to a square-root dependence with time (~*t*^−1/2^), characteristic of a diffusive type of transport (iii, Fig. 3A). Radial positions of individual swimmers exhibit large deviations from the population average, and swimmers begin to display chaotic behavior with large aperiodic oscillations (iii, Fig. 3A). Here, swimmers preferentially align along flow manifolds (iii, Fig. 3B). In the manifold trafficking regime (*p* = 5.1), the population radial displacement increases compared to that at *p* = 1.3 (iv, Fig. 3A) in accordance with the increased MSD values (Fig. 1B). The individual swimmer trajectories oscillate with larger amplitudes and shorter time scale, and swimmer alignment along manifolds is maximized (iv, Fig. 3B). Last, in the vortex trapping regime (*p* = 20), the population mean displacement is reduced once again (v, Fig. 3A). Individual swimmer trajectories continue to exhibit large chaotic deviations from the population mean, while a subset of swimmers become trapped in the initial quarter-cell. The alignment along flow manifolds is weakened compared to that at *p* = 5.1 (v, Fig. 3B).

To quantify the time evolution of swimmer alignment with flow manifolds, we compute the bias of probability density function (PDF) of θ along *k*π/2 as a function of *p* (see Fig. 3C and Materials and Methods). The bias is defined as the PDF normalized by that of a random uniform distribution. While no orientational bias occurs in the free-swimming regime (*p* = 0.13), we find weak swimmer depletion from manifolds at the onset of flow-induced transport; note that the PDF bias falls below 1 for *p* = 0.5. As the flow strength further increases to *p* = 5.1, swimmers become increasingly aligned with flow manifolds, reaching an order of magnitude increase in the probability bias before ultimately decreasing at the maximum *p* = 20. Swimmer aggregation dynamics show similar trends (Fig. 3D). Swimming particles become increasingly aggregated toward flow manifolds as *p* increases, reaching an order of magnitude increase in probability at *p* = 5.1 before decreasing at *p* = 20 due to trapping at the vortex core. Overall, we find that the deterministic-to-chaotic transition in individual cell trajectories underlies the flow-induced enhancements in transport at the population level. The collective convergence toward and alignment with flow manifolds enable subsequent chaotic trafficking by the time-periodic flow.

### An optimal dispersion regime occurs at intermediate pathlengths

Flow-induced transport can influence the ability of microorganisms to explore the flow domain and forage for nutrients. The ability to cover larger flow regions (or volumes) is conducive to nutrient access, whereas trapping results in rapid nutrient depletion. Here, we quantify this behavior by monitoring the statistics of unique quarter-cells traveled by the swimmers during dispersion as a function of flow pathlength *p* (Fig. 3E). In the free-swimming regime *p* = 0.13, the PDF distribution shows a narrow peak around a small set of quarter-cells. These quarter-cells locate along the swimmer trajectory, as shown by the linear increase in the number of unique quarter-cells traveled with time (i, Fig. 3F). As flow-induced transport commences (0.51 < *p* < 5), the distribution of unique quarter-cells traveled widens and shifts to higher values (Fig. 3E). This result indicates that swimmers are increasingly able to explore a larger set of vortex cells as *p* increases. In addition, the time development of the statistics increasingly follows a square-root relationship (ii to iv, Fig. 3F), consistent with the diffusive nature of the flow-induced transport. This enhancement effect is maximized at *p* = 5.1 in the manifold trafficking regime (Fig. 3, E and F, iv) before decreasing drastically in the vortex trapping regime (*p* = 20), where a large fraction of cells become confined to a single vortex cell and the distribution shifts to lower values (Fig. 3, E and F, v). Hence, the time-periodic flow maximally increases bacterial dispersion in terms of the number of unique vortex cells traveled in the manifold trafficking regime.

### Slow swimmers display elevated flow-induced transport relative to the free-swimming regime

**Fig. 4.**
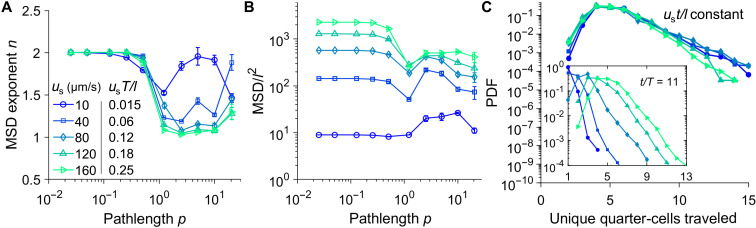
Effect of swimmer swimming speed on swimmer transport by time-periodic flow. (**A**) The scaling exponent *n* of the stroboscopic MSDs of active swimmers as a function of flow pathlength *p* for various swimming speeds. Error bars represent SDs from three replicates. (**B**) Long-term MSD normalized by quarter-cell length *l*^2^ at *t*/*T* = 200 as a function of flow pathlength *p* for various swimming speeds. (**C**) Distribution of unique quarter-cells traveled by the swimmers at identical time points scaled by swimming speed *u*_s_ and flow cell width *l* for pathlength *p* = 1.3. Inset: Same distribution at the identical unscaled time points.

We now explore the effects of swimming speed variations on the flow-induced transport in each transport regime by systematically varying the nondimensional swimming speed *u*_s_*T*/*l*. We begin by noting that swimmer displacements under flow-induced transport are diffusive in nature for *p* ≳ 0.5 for all swimming speeds investigated (Fig. 4A); the exception is for very slow swimmers *u*_s_ = 10 μm/s (*u*_s_*T*/*l* = 0.015), which will be discussed below. Here, we explore how flow-induced trafficking can lead to markedly different transport efficiencies (measured by MSD/*l*^2^) relative to the free-swimming case. First, for swimmers with speed comparable to that of *V. cholerae* (*u*_s_*T*/*l* = 0.12; Fig. 4B), the flow-induced transport in the manifold trafficking regime (*p* = 5) is on par with that in the free-swimming case (*p* < 0.5). Slower swimmers (*u*_s_*T*/*l* = 0.015 to 0.06; Fig. 4B), however, experience a significant boost in transport in the manifold trafficking regime (*p* = 5) relative to the free-swimming case (*p* < 0.5). On the other hand, the transport of faster swimmers (*u*_s_*T*/*l* = 0.18 to 0.25; Fig. 4B) is strongly hindered by the flow relative to free swimming. These results show that slow-swimming microorganisms may exploit time-periodic flows to achieve efficient dispersion even without large energy expenditures on motility.

### Swimming speed affects dispersion rate but not the patterns in the manifold confinement regime

For smooth swimmers in quiescent flow (i.e., free swimming), an increase in swimming speed linearly reduces the travel time required for a given displacement. Here, we explore whether such linear scaling holds in the flow-induced transport regimes. To this end, we monitor the statistics of the unique quarter-cells traveled at various times and swimming speeds. In the manifold confinement regime (*p* = 1.3) where the reduction in transport is maximal, we find that, for a fixed time interval (e.g., *t*/*T* = 11), faster swimmers traverse a larger number of flow cells (Fig. 4C, inset). We can further collapse all the curves to an identical distribution via a speed-compensated time, *u*_s_*t*/*l* (Fig. 4C), suggesting that in the manifold confinement regime, the time needed to reach a given level of dispersion simply reduces linearly for fast swimmers. The collapse into a universal curve is also observed for the swimmer alignment and aggregation toward flow manifolds (fig. S5). Furthermore, by linearly compensating time with swimming speed, such that *u*_s_*t*/*l* is constant, we find that swimmer dispersion patterns superimpose with one another (fig. S6, A to H). The dispersion patterns for *u*_s_*T*/*l* = 0.06 in the first row (fig. S6, A to D) are equivalent to that at identical scaled times in the second row (fig. S6, E to H) for different swimming speeds. We can conclude that while swimmer speed controls the rate of dispersion, speed apparently does not alter the dispersion pattern or the dynamics in the manifold confinement regime.

### Swimmers engage in chaotic escape spirals with exponential scaling in time before trafficking along manifolds

**Fig. 5.**
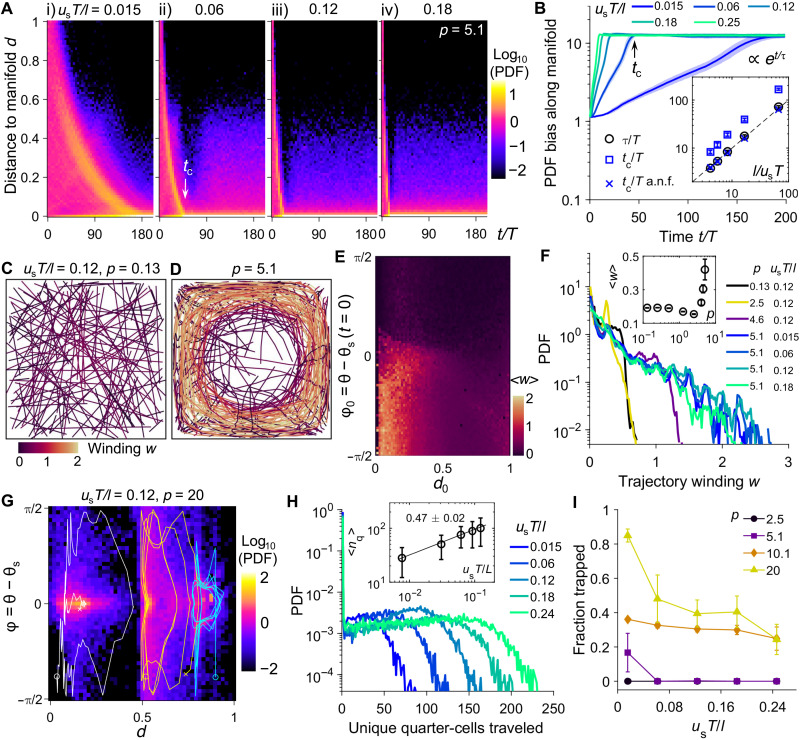
Effect of swimming speed on transport dynamics in the manifold trafficking and vortex trapping regimes. (**A**) PDF distribution of swimmer distance to flow manifolds *d*, in the manifold trafficking regime *p* = 5.1, for (i) *u*_s_*T*/*l* = 0.015, (ii) *u*_s_*T*/*l* = 0.06, (iii) *u*_s_*T*/*l* = 0.12, and (iv) *u*_s_*T*/*l* = 0.18. White arrow indicates the critical time *t*_c_ to reach flow manifolds. (**B**) PDF bias of swimmer aggregation toward flow manifolds for various values of *u*_s_*T*/*l*. Black arrow indicates the critical time *t*_c_. Inset: Normalized τ/*T* of the exponential growth, the critical time *t*_c_/*T*, and the critical time of arrival without flow *t*_c_/*T* a.n.f., versus inverse scaled swimming speed *l*/*u*_s_*T*. Dashed line: The abscissa is equal to the ordinate. (**C** and **D**) Swimmer trajectories before reaching flow manifolds *u*_s_*T*/*l* = 0.12 for (C) *p* = 0.13 and (D) *p* = 5.1. Colors indicate trajectory winding *w*. (**E**) Population-averaged winding <*w*> before reaching flow manifolds, in the space of swimmer initial distances to flow manifolds, *d*_0_, and orientations relative to the flow streamlines ϕ_0_ = θ − θ_s_. Negative ϕ_0_ implies swimming toward the vortex core, and positive implies swimming away. (**F**) Distribution of *w* for various pathlengths and swimming speeds. Inset: Population-averaged winding <*w*> for *u*_s_*T*/*l* = 0.12. (**G**) Final swimmer distribution in the transformed parameter space (*t*/*T* = 200, *u*_s_*T*/*l* = 0.12, and *p* = 20). Representative trajectories from the start (circle) to the end time (cross) are shown. (**H**) Swimmer unique quarter-cells traveled at *p* = 20 for various swimming speeds. Inset: Mean number of unique quarter-cells traveled for nontrapped cells. The line indicates the power-law fit. (**I**) Fraction of trapped swimmers versus swimming speed. All shaded regions and error bars indicate SDs from three replicates.

The simple linear scaling between swimming speed and the rate of dispersion breaks down for stronger flows (*p* ≥ 5). We first monitor the time development of the distribution of swimmer nondimensional distance to flow manifolds, *d* = cos(2π*x*/*L*) cos(2π*y*/*L*), where *d* = 1 at the vortex core and *d* = 0 at the boundaries between quarter-cells (see Materials and Methods). In the manifold trafficking regime where swimmer transport is maximally enhanced (*p* = 5.1; Fig. 5A), we find that for swimming speeds (*u*_s_*T*/*l*) ranging from 0.015 to 0.18, most swimmers converge toward flow manifolds as *d* decreases to zero; that is, active swimmers are attracted to the unstable flow manifolds. The rate of convergence is exponential in time for all swimming speeds (i to iv, Fig. 5A). The local peak in the PDF reaches zero at a critical time *t*_c_ (ii, Fig. 5A) after which the distribution reaches a stationary state where most swimmers are on flow manifolds. The exponential scaling implies a slowdown in the rate of convergence as swimmers approach flow manifolds. The characteristic time τ of the exponential convergence toward flow manifolds, exp(*t*/τ), can be observed in the PDF bias (Fig. 5B). As swimming speed increases, the characteristic time scale τ and the critical time *t*_c_ increase linearly with *l*/*u*_s_ (Fig. 5B, inset). Since slow swimmers (*u*_s_*T*/*l* = 0.015) remain in the spiral through the simulation, the MSD scaling exponent is approximately 2 (ballistic behavior) in the trafficking regime. We note that a plateau value in PDF bias is reached and is independent of the swimming speed, where approximately 10% of the swimmers remain outside of flow manifolds (fig. S7).

We next explore the origin of the exponential aggregation that occurs toward flow manifolds. In the trivial case of free swimming in quiescent flow, swimmer arrival at flow manifolds is linear in time for smooth swimmers initially in the vortex interior (fig. S8) and the critical time, *t*_c_ a.n.f. (arrival no flow), is equal to *l*/*u*_s_ or the time to reach *d* = 0 via straight trajectories (*t*_c_ a.n.f. inset, Fig. 5B). Hence, swimmers in the manifold trafficking regime must display additional flow-induced dynamics leading to the exponential slowdown. To demonstrate this behavior, we plot the stroboscopic swimmer trajectories colored by the unsigned winding number *w* around the elliptic point of the vortex (see Materials and Methods) for both the free-swimming (*p* = 0.13; Fig. 5C) and the manifold trafficking (*p* = 5.1; Fig. 5D) regimes. In the *p* = 0.13 case, swimmer trajectories are straight with minimal winding around the vortex center before reaching flow manifolds (Fig. 5C). In the *p* = 5.1 case, however, a subset of swimmer trajectories spiral multiple times around the vortex center as they approach flow manifolds (Fig. 5D). The drastic increase in winding, which is absent near the elliptic point, leads to the exponential slowdown in convergence.

The conditions that give rise to the spiraling escape toward the flow manifolds are now explored. To this end, we compute the ensemble-averaged trajectory winding <*w*> before reaching flow manifolds for swimmers with different initial distances *d*_0_ and initial orientation angles relative to local streamline tangent ϕ_0_ = θ − θ_s_ at *t* = 0 (Fig. 5E). The sign convention of ϕ is such that zero indicates alignment with the local streamline, negative indicates alignment toward the flow manifolds, and positive indicates alignment toward the elliptic points (see Materials and Methods). This parameter space allows us to directly monitor swimmer spatial distribution relative to flow manifolds and the swimmer’s tendency to approach or avoid such flow structures. We find that swimmers that are initially near flow manifolds *d*_0_ ≲ 0.2 but swim toward the elliptic point (ϕ_0_ < 0) or parallel to streamline (ϕ_0_ ≈ 0) have high winding. By contrast, swimmers that are initially near the vortex core (*d*_0_ ≳ 0.5) or that swim outward (ϕ_0_ > 0) have low winding. Typical trajectories in the (*x*, *y*) plane for swimmers that initially swim away from the elliptic point, parallel to the streamline, and toward the elliptic point (fig. S9) are consistent with these swimmer spiraling conditions. Unexpectedly, swimming speed variations do not alter trajectory winding during escape. Fast swimmers do not experience less winding relative to slow swimmers, and the population distribution of trajectory winding *w* before reaching flow manifolds is independent of swimming speed (Fig. 5F). Rather, the increase in flow strength triggers the rapid increase in <*w*> and dictates the degree of winding that will occur in the manifold trafficking regime (Fig. 5F, inset).

### Faster swimming facilitates escape from chaotic limit cycles in the vortex trapping regime

To probe the mechanism underlying vortex trapping for strong flows, we monitor the swimmer cell fate in the parameter space of (*d*, ϕ) or distance to flow manifolds and the angular deviation from local streamlines. The swimmer distribution at long times for *p* = 20 reveals the emergence of three trapping islands each centered around ϕ = 0 but spanning different *d* values (Fig. 5G). Stroboscopic swimmer trajectory in each region displays a chaotic limit cycle that dwells at fixed *d* values and near ϕ ≈ 0 (aligning with the streamlines) for extended times. These swimmers stay within the chaotic island without penetrating the transport barriers (at critical *d* values) into neighboring regions. However, occasionally, these swimmers undergo large excursions in *d* and deviations from alignment with the flow. The chaotic stroboscopic swimmer trajectories in the (*x*, *y*) space are shown in fig. S10. We note that swimmers that are initially near flow manifolds and are swimming away from the elliptic point do not get trapped. They nonetheless participate in the flow-enabled trafficking and explore a large number of flow cells (Fig. 5H).

Last, faster swimmers have significant transport advantages over slow swimmers in the vortex trapping regime. First, an increase in swimming speed at *p* = 20 results in a larger number of flow cells traveled (Fig. 5H) where the population-level dispersion <*n_q_*> scales with the square root of the swimming speed (Fig. 5H, inset). Second, faster swimmers are also better at escaping vortex trapping than slower swimmers (Fig. 5I). Phase diagrams for the flow-induced swimmer transport and trapping as functions of both swimming speed and flow pathlength are summarized in fig. S11. Hence, in the strongest time-periodic flow, swimmers are locked in their alignment and separation from flow manifolds and orbit in chaotic limit cycles. Faster swimmers, however, can escape flow-induced trapping and continue to explore the flow domain.

## DISCUSSION

Microorganisms often encounter unsteady flows both in nature and in industrial applications. The coupling between motility and flow is critical to the dispersion, reproduction, and overall survival of microswimmers. In oceans, rivers, and bioreactors, unsteady flows with strong local velocity gradients are present across a large range of time scales and length scales. While swimmer transport has been extensively studied in steady flows of vortex and convection roll arrays, here, we explore the chaotic and nonmonotonic transport of microswimmers via a model time-periodic mixing flow using both Langevin simulations and bacterial dispersion experiments. Two nondimensional parameters control the active swimmer transport in the unsteady flow, namely, the pathlength, which sets the nature of the background flow, and nondimensional swimming speed relative to the flow. For fixed-speed swimmers with minimal Brownian effects, increasing the pathlength parameter revealed three distinct flow-enabled transport regimes with notably different dispersion dynamics. At moderate flow strength relative to swimming, the swimmers are increasingly attracted toward the boundaries of the flow cells, leading to a marked reduction in MSD and strong weakening of ballistic swimming. As pathlength further increases, a distinct flow-induced transport emerges that rapidly disperses swimmers in a chaotic and purely diffusive fashion along paths formed by vortex boundaries. To participate in the chaotic trafficking along vortex boundaries, however, a subset of swimmers need to escape the vortex via exponential spirals that are universal for swimmers, fast or slow. Facilitated by the chaotic transport along flow manifolds, the number of unique flow cells traversed by swimmers during dispersion is maximized, leading to the largest exposure to potential foraging grounds in the flow domain. Such enhancements do not extend indefinitely, however, as further increases in flow strength result in the failure of swimmers to escape the vortex. Instead, swimmers are locked in chaotic limit cycles within the vortex. The optimal dispersion emerges due to the competition between the chaotic transport at the vortex boundaries and the strong reorientation and trapping at the vortex core. Faster swimmers progressively overcome flow-induced trapping and regain efficient dispersion in this flow regime. Our results show that, although the swimmer displacement due to flow alone is fully reversed in any given cycle, time-periodic flows can still fundamentally alter the transport outcome and dispersion of the swimmer via the nonlinear and chaotic coupling between flow time scale, velocity, and swimmer motility.

The two nondimensional control parameters may be generalized beyond the specifics of the flow system here to underscore active matter transport in unsteady flows for smooth swimmers with a range of shapes, speeds, and rotational diffusivities. Our results will be especially relevant for synthetic active colloids such as Janus-like particles lacking inherent rotational diffusivity, fast swimmers where rotational reorientation is weak compared to ballistic swimming, and bacterial species that do not run and tumble, such as smooth-swimming *Bacillus subtilis* and chemotaxis mutants such as Δ*cheY* mutants for *Escherichia coli* and Δ*cheY3* mutants in *V. cholerae* (*43*).

We note several limitations for the current study. We do not consider swimmers with frequent reorientations. Second, our model treats swimmers as point particles that do not locally modify the imposed flow. Therefore, we cannot accurately capture the transport behavior of extremely dense microbial suspensions, where steric interactions, finite size effect, and feedback to the flow are pronounced. In regions of sharp velocity gradient, such as hyperbolic points and flow manifolds, a small variation in swimmer position due to length could lead to substantial variations in swimmer trajectory. Last, experimentally obtaining high-resolution long-term MSD and MSD scaling exponents for micrometer-sized bacteria over centimeter-sized flow cells, especially under strong imposed flow gradients, represents a substantial challenge, which we seek to address in future works.

We hypothesize that confinement at flow manifolds at moderate flows and trapping in the vortex cores at strong flows for extended times may lead to increased swimmer encounter rates, emergent gradients in nutrients and chemoattractants, and local accumulation of quorum-sensing signaling molecules in natural settings. Alterations in these biological stimuli rendered by the time-periodic flow may have significant implications in dictating transitions in microbial lifestyles and survival strategies. Flow-induced dispersion is most evident for slow swimmers compared to free swimming in quiescent flow. Hence, by careful selection and control of flow structure and pathlength, one can devise an optimal transport strategy to maximize microbe dispersion in time-periodic flow systems such as mixing bioreactors, especially for swimmers with limited motility. Furthermore, by exploiting the vortex trapping regime at high pathlength, where faster swimmers escape the initial flow cell much quicker than slow swimmers, one could design tunable devices to preferentially sort swimmers by speed. Our results on the nonlinear coupling between flow structure, time periodicity, and swimmer transport could also have implications for the lifestyle transitions and fates of small marine organisms.

## MATERIALS AND METHODS

### Langevin simulation

A time-periodic 2D Taylor-Green vortex type of flow is used in the Langevin simulation. In the (*x*, *y*) plane, the stream function ψ of the flow is

$$\psi =\frac{UL}{2\pi }\cos(2\pi ft)\cos(2\pi x/L)\cos(2\pi y/L)$$ (1)

where *U* is the peak flow velocity at flow manifolds, *f* is the frequency of the flow, *L* = 2*l* is the wavelength of the periodic flow cell that consists of four quarter-cells, and *l* = 6.5 mm is the width of the quarter-cell. The peak vorticity at vortex cores is ω = 2π*U*/*l*. The velocity field is then ***u*** = < ψ*_y_*, − ψ*_x_*>, where *x*, *y* in subscript denote partial differentiation. In addition, we define a nondimensional flow pathlength parameter *p* = *UT*/2π*l* = ω/4π^2^*f*, which is the maximum angle swept by the flow near the vortex core in a cycle, normalized by a full circle. The nondimensional pathlength is a general feature of the time-periodic flow.

We model the swimmers as active ellipsoids moving along their major axis (*31*, *47*), whose orientation angle with + *x* axis is given by θ. The aspect ratio of the swimmer is γ (γ = 5 for *V. cholerae* unless otherwise stated) and the geometry parameter α = (γ^2^ − 1)/(γ^2^ + 1). The swimming speed is *u*_s_ (*u*_s_ = 80 μm/s for *V. cholerae* unless otherwise stated). The swimmers are advected by the flow field and reoriented by the shear and rotational flow gradients

$$dq=\left[\frac{\nabla u-\nabla u^{T}}{2}+\alpha \frac{\nabla u+\nabla u^{T}}{2}\cdot (I-q⊗q)\right]q\;dt$$ (2a)

$$dx=(u+u_{s}q)dt+\sigma_{s}dW$$ (2b)

where **q** is the unit director of the swimmer, **x** = 〈*x*, *y*〉 is the position vector of the swimmer, ∇***u*** is the flow vorticity tensor, **W** = 〈*W_x_*, *W_y_*〉 is the Wiener noise, and *W_x_*, *W_y_* represent the stochastic Wiener noise in the corresponding variables. We include translational diffusion in lateral *x*, *y* directions where the Wiener noise follows a Gaussian distribution with SD σ_s_. Note that the translational diffusion due to Brownian motion is $D_{0}=\sigma_{s}^{2}/n_{s}$, where *n*_s_ = 2 is the dimension of the system and is estimated by the Stokes-Einstein relation with the geometrically averaged swimmer dimensions (ellipsoid 1.7 μm in length and 0.3 μm in width) for simplicity. This amounts to *D*_0_ = 4.0 × 10^−13^ m^2^/s in water-like viscosities. We note that the level of displacement due to Brownian motion is much less than that of swimmer motility for motile swimmers. We incorporate finite swimmer rotational diffusivity, as given by $D_{r}=\sigma_{r}^{2}$ and *W*_θ_. Expressed in terms of the flow stream function, the orientation angle θ and position *x*, *y* of the swimmer are governed by

$$d\theta =\left[-\frac{\psi_{xx}+\psi_{yy}}{2}+\alpha \left(\frac{\psi_{yy}-\psi_{xx}}{2}\cos2\theta -\psi_{xy}\sin2\theta \right)\right]dt+\sigma_{r}{dW}_{\theta }$$ (3a)

$$dx=(\psi_{y}+u_{s}\cos\theta )dt+\sigma_{s}{dW}_{x}$$ (3b)

$$dy=(-\psi_{x}+u_{s}\sin\theta )dt+\sigma_{s}{dW}_{y}$$ (3c)

The spatial domain of the simulation is unbounded. For each replicate of a set of flow conditions, 10^4^ swimmers are initially seeded uniformly and randomly in the quarter-cell from *x*/*l* = −0.5 to 0.5 and *y*/*l* = −0.5 to 0.5 with random swimming directions. Three replicates are conducted for each set of flow conditions. A second-order Runge-Kutta scheme is used with a marching step of 10^−4^ s to a final time of *t*_f_ = 2000 s. The marching step is determined by minimizing the numerical error in displacements for passive nondiffusive particles in the flow.

By defining $\tilde{x}=x/l,\;\tilde{y}=y/l,\;\tilde{t}=t/T,\;\tilde{\psi }=\psi T/l^{2},\;{\tilde{u}}_{s}=$ $u_{s}T/l,\;\mathrm{and}\;{\tilde{\sigma }}_{s}=\sigma_{s}/l$, the governing equation can be nondimensionalized as

$$\tilde{\psi }=2p\cos(2\pi \tilde{t})\cos(\pi \tilde{x})\cos(\pi \tilde{y})$$ (4a)

$$d\theta =\left[-\frac{{\tilde{\psi }}_{\tilde{x}\tilde{x}}+{\tilde{\psi }}_{\tilde{y}\tilde{y}}}{2}+\alpha \left(\frac{{\tilde{\psi }}_{\tilde{y}\tilde{y}}-{\tilde{\psi }}_{\tilde{x}\tilde{x}}}{2}\cos2\theta -{\tilde{\psi }}_{\tilde{x}\tilde{y}}\sin2\theta \right)\right]d\tilde{t}+\sigma_{r}{dW}_{\theta }$$ (4b)

$$d\tilde{x}=({\tilde{\psi }}_{\tilde{y}}+{\tilde{u}}_{s}\cos\theta )d\tilde{t}+{\tilde{\sigma }}_{s}{dW}_{x}$$ (4c)

$$d\tilde{y}=(-{\tilde{\psi }}_{\tilde{x}}+{\tilde{u}}_{s}\sin\theta )d\tilde{t}+{\tilde{\sigma }}_{s}{dW}_{y}$$ (4d)

where swimmer transport dynamics are controlled by pathlength *p* and nondimensional swimming speed ${\tilde{u}}_{s}$. We note that in the limit of *p* → 0, swimming dominates, whereas for *p* $\gg {\tilde{u}}_{s}$, the effect of swimming will dominate only in regions where ${\tilde{\psi }}_{\tilde{x}}$ and ${\tilde{\psi }}_{\tilde{y}}$ are small, i.e., near the vortex core and quiescent time phases. The boundary of these regions will depend on the competition between flow *p* and swimming ${\tilde{u}}_{s}$.

### Swimmer statistics

Long-term MSDs are computed by ensemble averaging the MSDs for all swimmers at the final time *t*_f_ = 2000 s or 200*T*. The long-term MSD scaling exponent *n* is computed by a power-law least-square fitting from *t*/*T* = 50 to 200. The nondimensional distance to flow manifolds is defined by *d* = cos (2π*x*/*L*) cos (2π*y*/*L*) and is 1 at the vortex core and 0 at flow manifolds, i.e., boundaries between quarter-cells. The PDF bias along flow manifolds is computed as follows: At a given time, the swimmer probability for *d* < 0.03 is evaluated and normalized by the probability for *d* < 0.03 if all swimmers were uniformly distributed in space. The PDF bias along *k*π/2 is defined similarly where the probability of swimmer orientation is aligned with flow manifolds or falls within |θ − *k*π/2| ≤ 0.035, *k* = ±2, ±1, and 0, and is normalized by that if all swimmer orientations are uniformly distributed. The trajectory winding number before reaching flow manifolds *w* is calculated as the net angle traversed around the elliptic point of the seeding flow cell, from *t* = 0 to the time when the swimmer reaches *d* < 0.03, normalized by the full circle 2π. The winding number is unsigned; i.e., clockwise or counterclockwise winding angles are not differentiated. Population-averaged winding is denoted as <*w*>. Swimmer orientation relative to the tangent of the local flow streamlines is defined as the acute angle formed between the swimmer orientation vector and the tangent line, ϕ = θ − θ_s_, and is offset to the range −π/2 ≤ ϕ ≤ π/2. The angle of the streamline tangent is θ_s_ = atan2(−ψ*_x_*, ψ*_y_*). The sign convention of ϕ is such that zero indicates alignment with the local streamline, negative indicates alignment toward flow manifolds, and positive indicates alignment toward the elliptic points.

### Bacterial strain and suspension preparation

We use the comma-shaped Gram-negative bacterium *V. cholerae* as the active swimmer in the bacterial dispersion experiments. The *V. cholerae* strain used (C6706, LZV918) is defective in virulence (Δ*tcpA*) and constitutively produces the green fluorescent protein. Bacteria were grown overnight at 37°C with shaking in LB medium supplemented with streptomycin (30 μg/ml). *V. cholerae* cells were collected by centrifugation and resuspended at the original volume in phosphate-buffered saline (PBS; 1×) before use in experiments.

### Flow cell apparatus

The experimental flow cell apparatus consists of a periodic array of permanent magnets, arranged with alternating polarity to fill a 10 cm by 10 cm chamber at a uniform spacing, or half-period, of *l* = 6.5 mm. Each magnet has a diameter of 4.8 mm. A thin fluid layer (2 mm thick) of electrolyte solution (1× PBS) fills the flow chamber and is placed in between two graphite electrodes on opposite sides of the flow chamber. By imposing a sinusoidal voltage across the electrodes, we generate magnetohydrodynamic forcing in the fluid above the permanent magnets. The voltage amplitude ranges from 0.5 to 4 V, and the frequency ranges from 0.05 to 0.2 Hz. This Lorentz body force, induced by the combination of the spatially ordered array of magnets and time-dependent current, drives the spatially and temporally periodic vortices. Typical peak flow velocities *U* range from 0.02 to 2 mm/s. The Reynolds number of the flow, defined by *Re* = *Ul*/ν, based on quarter-cell width *l* and fluid kinematic viscosity ν, ranges from 0.1 to 10. The Peclet number, defined by *Pe* = *Ul*/*D*_0_, is larger than 10^5^ under all conditions. The experimental flow field and vorticity are similar to those of the model time-periodic Taylor-Green vortex in the simulations (fig. S1).

### Bacterial dispersion experiments

At the beginning of the experiment, fluid in the flow cell apparatus remains quiescent. A total of 50 μl of the fluorescent bacterial suspension is added to the center vortex of the flow chamber via an air displacement pipette. Immediately after addition, the voltage across the flow cell apparatus is activated. The camera images a region of approximately 2.5 cm × 2.5 cm at the center of the chamber at 30 fps. The flow chamber is illuminated by a series of light-emitting diode lights placed above it (470-nm peak emission wavelength). A barrier filter (530-nm peak bandpass filter) is placed in front of the camera to exclude excitation light. The fluorescent intensity, which correlates with bacterial cell density, is recorded for each pixel location. The duration of each experiment is around 10 min.

### Particle tracking velocimetry

To characterize the flow, we disperse 39-μm polystyrene particles on the surface of the fluid. The particle positions are imaged using a complementary metal-oxide semiconductor camera (IO Industries, Flare 4M180) operating at 30 fps. To compute flow velocity gradients, stroboscopic-averaged velocity fields, obtained by averaging particles at identical phases (60,000 data points per phase), are used. Particle tracking algorithms based on (*48*) are used to track tracer particle centroids. Briefly, the particle features were next identified using a local peak finder with filtering criterion, such as peak intensity, total intensity, radius of gyration, and aspect ratio. The centroids of the particles were refined using a Gaussian fit. To connect particles between consecutive frames, the mapping between particles that yielded the minimal total squared displacement was used and subsequently smoothed by a high-order polynomial.
