# Supplementary material for: Confinement, chaotic transport, and trapping of active swimmers in time-periodic flows
Source: Sci Adv. 2022 Dec 7;8(49):eadd6196. doi: 10.1126/sciadv.add6196 (PMC9728977; doi:10.1126/sciadv.add6196)
Supplement: Supplementary file 1 — Figs. S1 to S11 [file sciadv.add6196_sm.pdf]

Science Advances
AAAS

Supplementary Materials for

Confinement, chaotic transport, and trapping of active swimmers in time-periodic flows

Boyang Qin and Paulo E. Arratia

Corresponding author: Boyang Qin, bqin@princeton.edu; Paulo E. Arratia, parratia@seas.upenn.edu

Sci. Adv. **8**, eadd6196 (2022)
DOI: 10.1126/sciadv.add6196

The PDF file includes:

Figs. S1 to S11
Legends for movies S1 to S4

Other Supplementary Material for this manuscript includes the following:

Movies S1 to S4

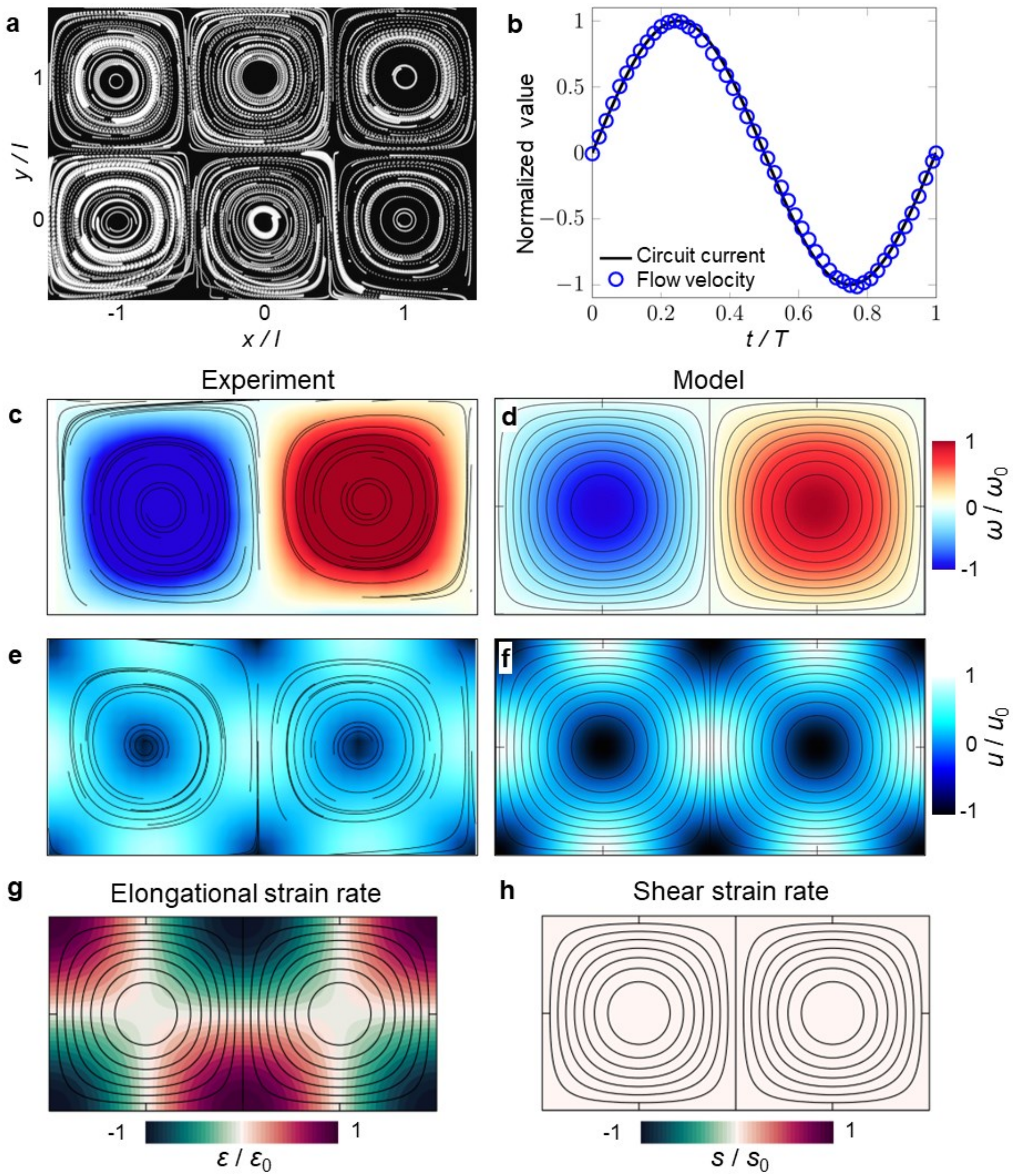

Fig. S1. Particle velocimetry of the flow in the experimental cell apparatus and comparison with the numerical model. (**a**) Tracer particle streak lines in the experimental flow cell. (**b**) Time dependency of the driving current and the field-averaged flow velocity, both normalized by the peak value. One period is shown. (**c**,**d**) Flow vorticity map, normalized by the peak value, overlaid with flow streamlines in (**c**) the experimental flow apparatus and (**d**) the numerical model. (**e**,**f**) Flow velocity magnitude map, normalized by the peak value, overlaid with flow streamlines in (**e**) the experimental flow apparatus and (**f**) the numerical model. (**g**,**h**) Flow elongational strain rate $\varepsilon = u_x - v_y$, and shear strain rate $s = u_y + v_x$ normalized by peak values in the model flow. Shear is minimal in the flow system.

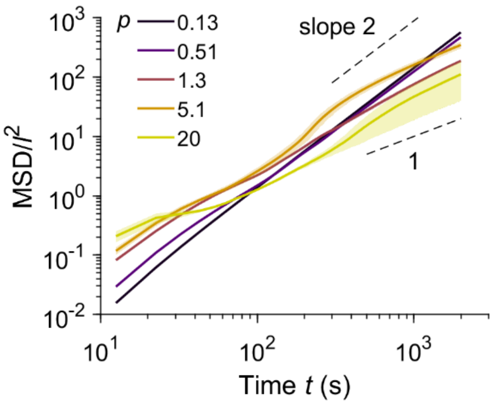

Fig. S2. Time development of mean squared displacements for various pathlength values. MSD normalized by quarter-cell length l^2 for passive particles without flow at t = 2000 s, for swimmers with speed u_s = 80 μm/s, $u_s T/l$ = 0.12. Dashed lines indicate the power law scaling relationships at the power shown. Shaded regions indicate standard deviations from three replicates.

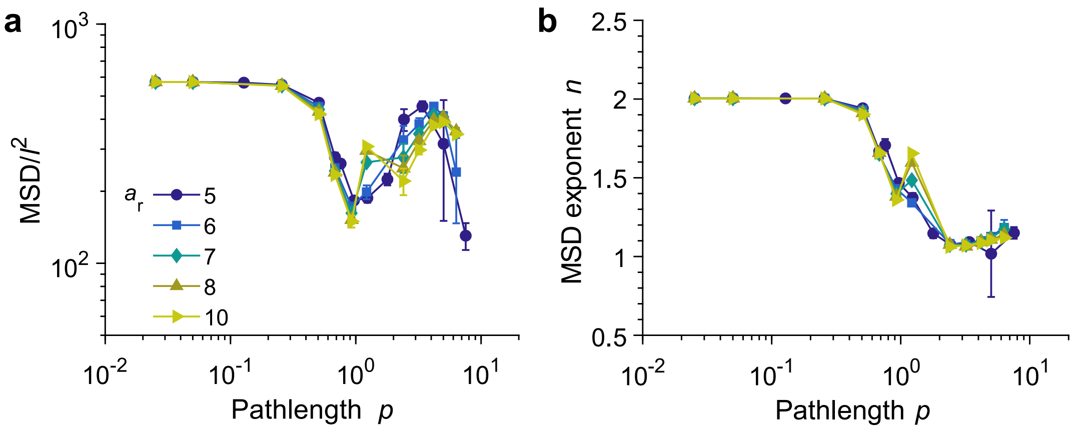

Fig. S3. The regimes of long-term transport of active swimmers in time-periodic flows are insensitive to shape variations for flagellar swimmers. (**a**) Ensemble-averaged long-term mean squared displacements (MSD) of active swimmers as a function of flow pathlength (p) for various swimmer aspect ratios at time $t = 2000$ s, normalized by the flow quarter-cell length l^2 for $u_s = 80$ μm/s or $u_s T/l = 0.18$. (**b**) The corresponding scaling exponent n of the ensemble-averaged stroboscopic MSD of active swimmers as a function of flow pathlength p.

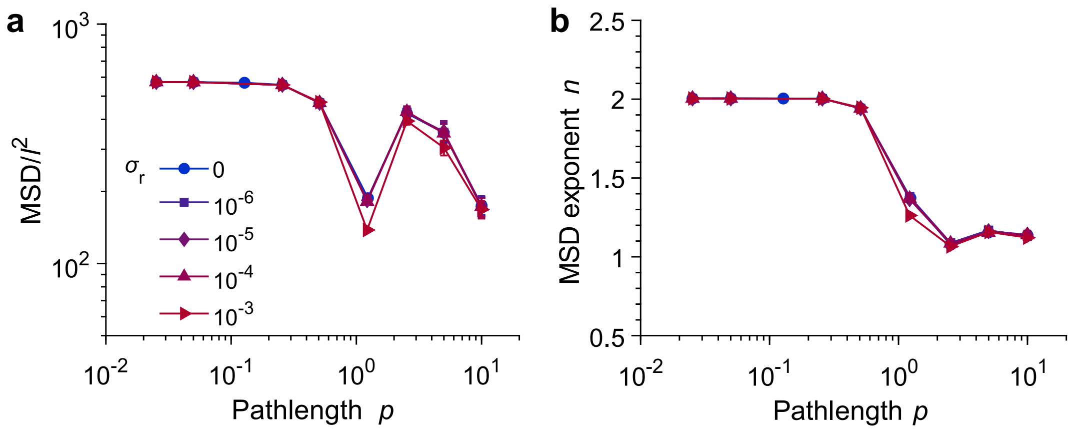

Fig. S4. The regimes of long-term transport of active swimmers in time-periodic flows are insensitive for smooth swimmers over a range of rotational diffusivity. (**a**) Ensemble-averaged long-term mean squared displacements (MSD) of active swimmers as a function of flow pathlength (p) for various swimmer rotational diffusivity (σ_r) at time t = 2000 s, normalized by the flow quarter-cell length l^2 for u_s = 80 µm/s or u_sT/l = 0.18. (**b**) The corresponding scaling exponent n of the ensemble-averaged stroboscopic MSD of active swimmers as a function of flow pathlength p.

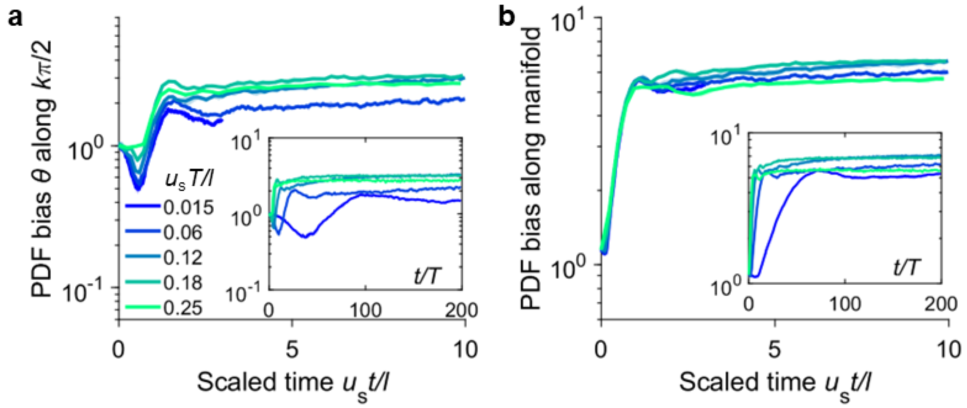

Fig. S5. Time development of swimmer alignment and aggregation toward flow manifolds collapses upon non-dimensional scaling of time with swimming speed. (**a**) PDF bias of swimmer orientation toward $k\pi/2$, $k = \pm 2, \pm 1, 0$, as a function of scaled time for various swimming speeds at p = 1.3. Shaded regions indicate standard deviations from three replicates. Inset: the identical PSD bias plotted against unscaled time t/T. (**b**) The PSD bias of swimmer aggregation toward flow manifolds as a function of scaled time for various swimming speeds at p = 1.3. Shaded regions indicate standard deviations from three replicates. Inset: the identical PSD bias plotted against unscaled time t/T.

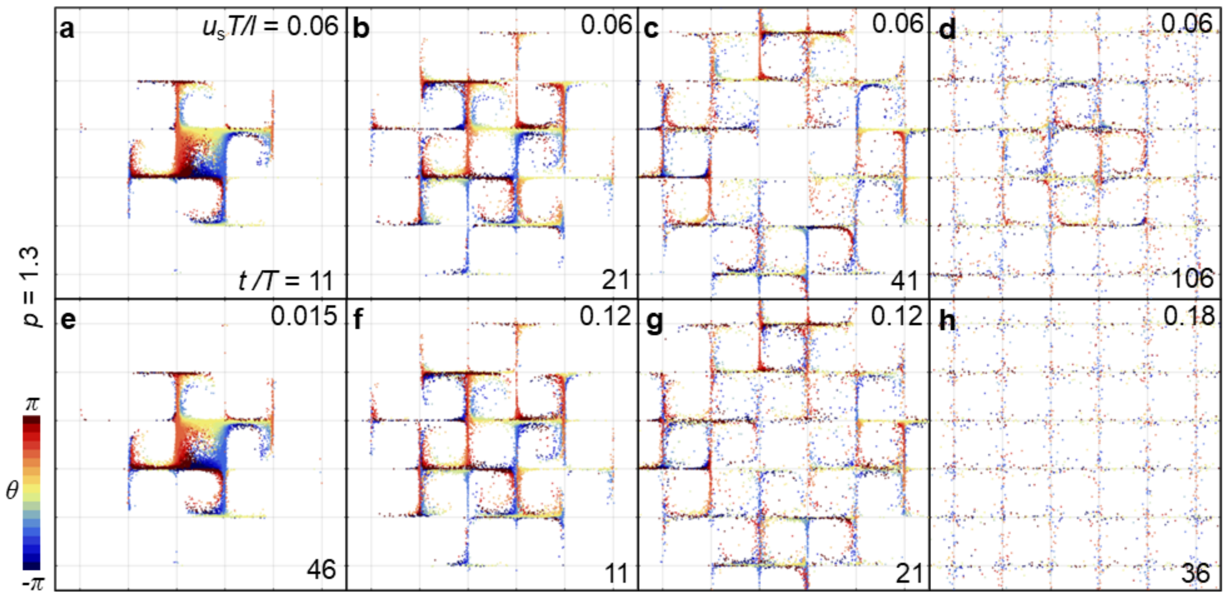

Fig. S6. Swimmer trajectory collapses upon non-dimensional scaling of time with swimming speed in the manifold confinement regime. (**a-d**) Swimmer locations colored by the corresponding swimmer orientation angles for swimming speed $u_s T/l$ = 0.06 and flow pathlength p = 1.3, in the manifold confinement regime, at the indicated time points. (**e-h**) The same transport patterns at the identical scaled time points for different swimming speeds corresponding to (**d-g**), at the same pathlength p = 1.3.

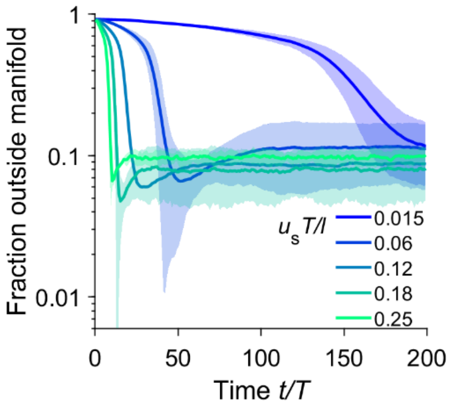

Fig. S7. Time development for the fraction of swimmers that are not on flow manifolds for pathlength p = 5.1 and various swimming speeds. The swimmer is on a flow manifold if $d \leq 0.03$. Shaded regions indicate standard deviations from three replicates.

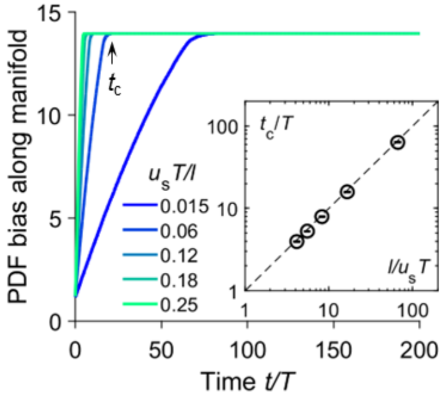

Fig. S8. Time development of the swimmer distribution bias toward flow manifolds in the free-swimming regime if the swimmer continues to remain at flow manifolds following its arrival. Various swimming speeds are shown for the quiescent flow case. The black arrow indicates the critical time t_c as shown in Fig. 5b. Shaded regions indicate standard deviations from three replicates. Inset: the normalized critical time as a function of scaled inverse velocity. The dashed line represents where the abscissa and the ordinate are equal.

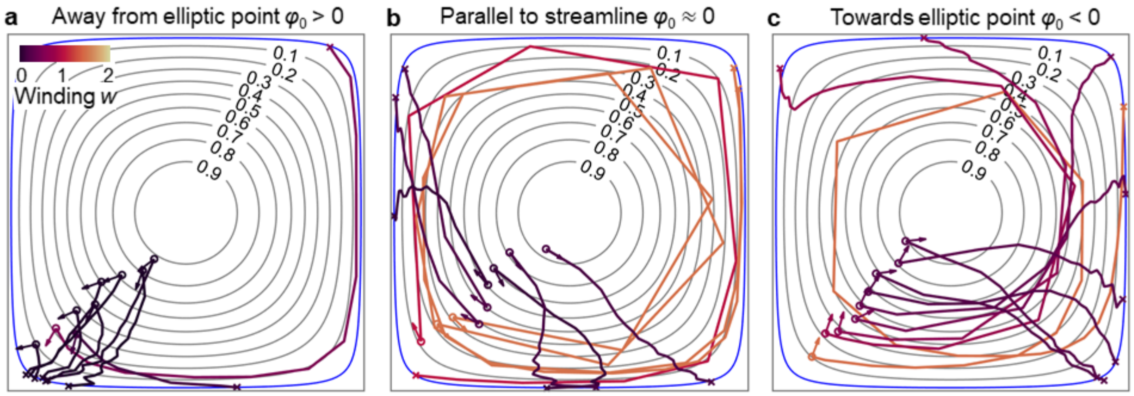

Fig. S9. Swimming parallel to streamlines or toward the elliptic point leads to high trajectory winding in the manifold trafficking regime. Stroboscopic swimmer trajectories prior to reaching flow manifolds at various starting positions and orientations at flow pathlength $p = 5.1$ for swimmers that initially swim (**a**) away from the elliptic point or $\varphi_0 > 0$, (**b**) parallel to the local streamlines $\varphi_0 \approx 0$, and (**c**) toward the elliptic point $\varphi_0 < 0$. Circles indicate the starting points of trajectories. Arrows indicate initial swimming directions. Crosses indicate swimmer positions when they reach flow manifolds. Colors indicate trajectory unsigned winding numbers w. Gray curves indicate contour lines for d.

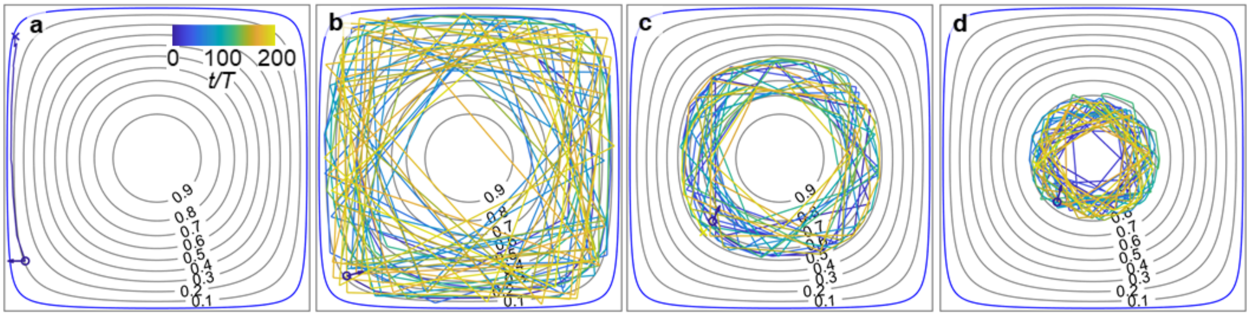

Fig. S10. Swimmers display distinct trapping outcomes for various initial conditions in the vortex trapping regime. Representative stroboscopic swimmer trajectories prior to reaching flow manifolds at various starting positions and orientations at flow pathlength $p = 20$ for a swimmer that initially (**a**) swims away from the elliptic point or $\varphi_0 > 0$ and is near flow manifolds $d_0 \approx 0.1$, (**b**) swims toward the elliptic point $\varphi_0 < 0$ and is near flow manifolds $d_0 \approx 0.1$, (**c**) swims toward the elliptic point $\varphi_0 < 0$ and is at an intermediate proximity relative to the elliptic point $d_0 \approx 0.5$ and (d) swims toward the elliptic point $\varphi_0 < 0$ and is near the elliptic point $d_0 \approx 0.8$. Circles indicate starting points of trajectories. Arrows indicate initial swimming directions. Crosses indicate swimmer positions when they reach flow manifolds. Colors indicate trajectory times t/T. Gray curves indicate contour lines for d.

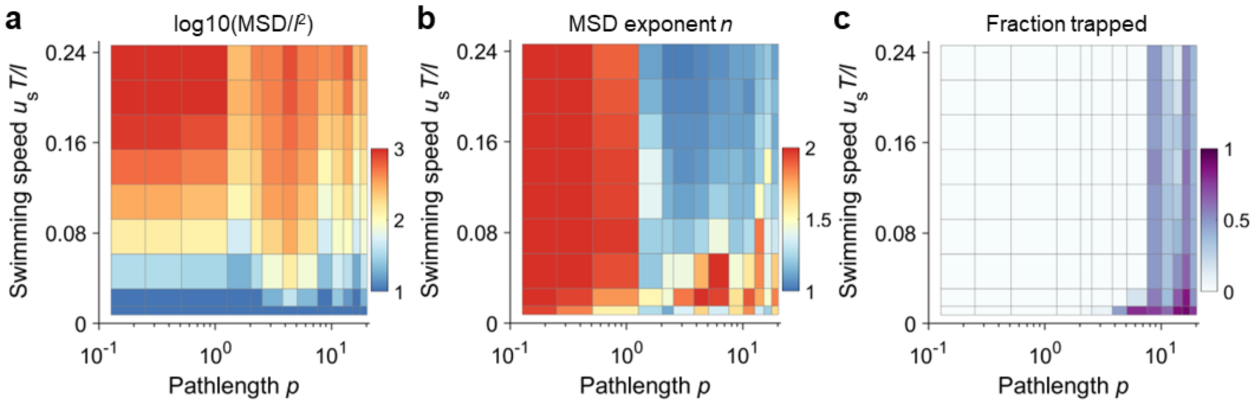

Fig. S11. Non-monotonic control of swimmer long-term transport and trapping. Phase diagrams of (**a**) long-term swimmer transport, (**b**) MSD scaling exponent n, and (**c**) the fraction of cells trapped at the flow cell core, in the parameter space of pathlength and swimming speed.

Movie S1. Stroboscopic swimmer trajectories (u_s = 80 µm/s, $u_s T/l$ = 0.12) in the time-periodic flow at the onset of the manifold confinement regime, p = 0.5. Colors denote swimmer orientation.

Movie S2. Stroboscopic swimmer trajectories (u_s = 80 µm/s, $u_s T/l$ = 0.12) in the time-periodic in the manifold confinement regime, p = 1.3. Colors denote swimmer orientation.

Movie S3. Stroboscopic swimmer trajectories (u_s = 80 µm/s, $u_s T/l$ = 0.12) in the time-periodic in the chaotic manifold trafficking regime, p = 5.1. Colors denote swimmer orientation.

Movie S4. Stroboscopic swimmer trajectories (u_s = 80 µm/s, $u_s T/l$ = 0.12) in the time-periodic in the vortex trapping regime, p = 20. Colors denote swimmer orientation.
